# Parents' perceptions of patient safety in paediatric hospital care—A mixed‐methods systematic review

**DOI:** 10.1111/jan.16361

**Published:** 2024-08-09

**Authors:** Maria I. Witkowska, Katja Janhunen, Natalia Sak‐Dankosky, Tarja Kvist

**Affiliations:** ^1^ Department of Nursing Science University of Eastern Finland Kuopio Finland; ^2^ Department of Clinical Nursing Medical University of Warsaw Warsaw Poland

**Keywords:** care, child nursing, family care, mixed method design, parenting, patient perspectives, patient safety, qualitative approaches, systematic reviews and meta‐analyses

## Abstract

**Aim(s):**

To identify and summarize evidence on paediatric patient safety in a hospital setting from parents' point of view.

**Design:**

A mixed‐methods systematic review.

**Prospero ID:**

ID number CRD42023453626.

**Data Sources:**

PubMed, Scopus, ScienceDirect, the Cochrane Library and the Wiley database were searched in July 2023.

**Review Methods:**

Two researchers independently applied eligibility criteria, selected studies and conducted a quality appraisal. Data‐based convergent synthesis and thematic content analysis were employed.

**Results:**

Twelve studies were included: eight qualitative research studies, two cross‐sectional studies, one non‐randomized experimental study and one mixed‐methods study. The results were grouped into two themes—parental perceptions of inclusion in paediatric patient safety and parental perceptions of exclusion from paediatric patient safety—and comprised seven main subthemes: comfort in communication, parental engagement, communication difficulties, withdrawal from activity, uncertainty about available information and threats to patient safety.

**Conclusions:**

Parents are willing to be engaged in care but require support from healthcare professionals, as they are often anxious about the condition of their children and actions they believe might be helpful. They need to be treated as valuable partners and be engaged in communication and decision processes.

**Impact:**

The development and implementation of interventions involving parents in ensuring the safety of hospitalized paediatric patients should be of the utmost priority to healthcare organizations, as the common theme throughout the included studies was the need for improved communication with and recognition of parents as allies.

**Reporting Method:**

The Preferred Reporting Items for Systematic Reviews and Meta‐Analysis (PRISMA) checklist was followed.

**Patient or Public Contribution:**

No Patient or Public Contribution.

**What does this paper contribute to the wider global clinical community?**

This paper provides the summary of the evidence on paediatric patient safety in the hospital setting from parents' point of view.

## 1 INTRODUCTION

The World Health Organization defines patient safety as ‘the absence of preventable harm to a patient and reduction of risk of unnecessary harm associated with health care to an acceptable minimum’ (World Health Organization, 2009). The need for the evolution of care, of which patient safety is one of six domains, has been pointed out since the early 2000s, when the first calls for change were made (Institute of Medicine (US) Committee on Quality of Health Care in America, 2001). Errors in medicine continue to occur, resulting in harm and causing the deaths of more than 3 million patients of all ages per year, with 50% of those cases being preventable (World Health Organization, 2023) and as many as one‐third of hospitalized children being affected by those errors (Mueller et al., 2019; Walsh et al., 2014). This has led to the quest to raise the level of patient safety around the globe while recognizing its challenges and prioritizing the implementation of improvement strategies (World Health Organization, 2021). Although recent times have increased the interest in paediatric patient safety, the number of sources on the topic remains limited and research priorities for paediatric patient safety have not yet been studied (Hoffman et al., 2019; Nicolì et al., 2022). Moreover, the relationship between patients' families and patient safety just recently began to be studied (Hoffman et al., 2019; Khan et al., 2016). There is no summary of the available research, which this review aims to address.

### 1.1 Background

Patient‐centred care can be described broadly as a healthcare practice that emphasizes treating each patient with the respect and dignity that each person inherently deserves, tailoring care to the individual needs of the patient and their family while applying evidence‐based practice (Sands & Sokol‐Hessner, 2017). Despite the general recommendation to implement patient‐centred care principles in clinical settings, institutions often minimize the effect of family members' inclusion. The family‐systems theory explains the relationships inside the family system as a whole: their subsystems as well as the individuality of each family member. It helps to understand each family's inner dynamics, the varying levels of closeness within the family, its boundaries and how the strength of the family together is greater than that of its parts individuals and subsystems (Allen, 2016). This knowledge helps to link parents and their experiences with those of their ailing children during hospitalization. They find themselves in a setting that is frequently, though not always intentionally, not family‐oriented, influencing the dynamic between the child and their parent. In the hospital environment, parents of the hospitalized children not only must perform daily duties and provide the semblance of normalcy but often have the task of translating their children's behaviour. They explain the symptoms and provide an exhaustive history of previous medical occurrences. They remain present and prepared for communicating with health care professionals and their diagnoses and treatment proposals, which are not always adjusted to the parents' level of health literacy (Brady et al., 2020; Karsenty et al., 2013).

The paediatric environment is especially prone to patient‐safety incidents, as children differ from adults both physically and mentally, for example, by requiring doses of medicine adjusted to their weight and are often too young to support and protect themselves. These differences require the adjustment of the healthcare provided, leading to heightened risks in medical care, most often regarding medication errors (Niemann et al., 2015; Rinke et al., 2014; Stang et al., 2018). The conscious and deliberate inclusion of parents, however, increases their involvement, eases their anxiety and helps to provide a sense of security to their children (de Melo et al., 2014) as well as ensure proper care and adherence to the needs of the patient (Brady et al., 2020).

It is crucial to acknowledge that it is plausible that families are experts on the subject of their offspring's health and that their child's wellbeing is their highest priority (Brady et al., 2020). While nurses, physicians and other healthcare specialists aim to provide excellent care, it is the parents who remain at the bedsides of their hospitalized children and can monitor them the most. As such, they might be the first to recognize a plausibly harmful incident or a nearing health decline (Hoffman et al., 2019). In the case of families with children with medical complexities, their capabilities to recognize the nuanced relationship between the underlying illness and acute infection sometimes stand in contrast to clinicians' more ‘textbook’ approach (Brady et al., 2020). Families are able to recognize between three to five times more adverse events and errors than are reported by hospital personnel, resulting in elevated safety‐detection rates and helping prevent their repetition (Khan et al., 2017). Family members should be allowed to engage in health processes as well as be aided with resources to raise their health literacy. Their ability to identify, create and implement practices for patient safety should be supported (Mueller et al., 2019) and it is recommended that healthcare professionals include this support in their practices, encouraging parental involvement in providing care (de Melo et al., 2014).

Due to the limited amount of research on the topic of paediatric patient safety and parental perspectives on it, an increase is recommended in the promotion of studies focusing on paediatric patients, including in the paediatric hospital setting (Nicolì et al., 2022). New policies and interventions are necessary, as well as further work on building a theoretical framework around patient safety for paediatric patients (Stang et al., 2018). The focus on educating and engaging health care specialists, patients and their families based on research and interventions should be expanded (Mueller et al., 2019). This systematic mixed methods review aims to describe the views of patient safety held by hospitalized children's parents. Despite the importance of this topic, there is not much literature on this issue available and not all studies met the inclusion criteria set by the authors for this review.

## 2 METHODS

### 2.1 Aims

The aim of this review was to identify and summarize the evidence on paediatric patient safety in the hospital setting from parents' point of view.

The addressed research question was: *What are parents' perceptions regarding paediatric patient safety in hospital care?*

### 2.2 Design

Given the nature of the main variable (parental perceptions), in this systematic review a mixed‐methods design was used to broaden the inclusion of studies (Gough, 2015) and address the research issue stated in the aim of the study (Pearson et al., 2015). It was reported according to the Preferred Reporting Items for Systematic Reviews and Meta‐Analysis (PRISMA) 2020 checklist (Page et al., 2021).

The protocol was registered with the PROSPERO register of systematic reviews in 2023, under the ID number CRD42023453626.

### 2.3 Search methods

First, the authors conducted a scoping search to identify the knowledge gap in the literature before starting this study and clarify three research questions. Next, the main search for this review was conducted in July 2023. The search strategy was based on an extensive literature search to identify relevant terms and their synonyms. The outcomes were also shared with an information specialist and applied in selected databases in various sequences: (‘patient safety’) AND synonyms of ‘pediatric’ AND ‘parent’ AND ‘view*.’ The complete search strategy is presented in Appendix S1.

### 2.4 Eligibility criteria

The inclusion criteria included peer‐reviewed studies published between 2013 and 2023 in English and available in PubMed, Scopus, ScienceDirect, the Cochrane Library or the Wiley database. The study design consisted of cross‐sectional studies, case–control studies, case reports, cohort studies, intervention studies, prevalence studies, qualitative research, quasi‐experimental studies and randomized controlled trials. Access to the full text was mandatory: either open access, access through the university's library or by sending the authors a direct request (in which case the threshold of 2 weeks for an answer was set). All studies had to focus on the experiences and views of parents and caregivers and their perceptions of patient safety during the hospitalization of their children.

Exclusion criteria comprised publications outside of the timeframe or unavailable in English. Reviews, secondary analyses and publications not concerning the selected subject were rejected. If a text was not available through open access or the library service was not granted in 2 weeks, it was also rejected. Two authors independently screened the publications for their eligibility.

### 2.5 Screening

**FIGURE 1:**
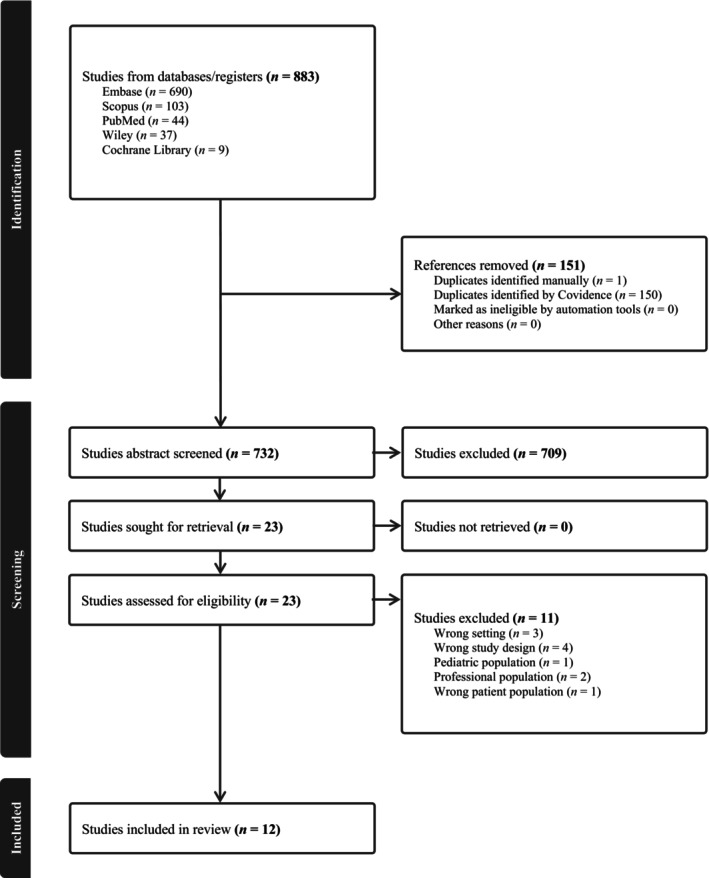
PRISMA flow diagram.

A total of 883 studies were included in the initial screening, of which 151 were duplicates. During abstract screening, 709 out of the remaining 732 studies were found to be irrelevant to the topic and were excluded. Of the 23 full‐text studies assessed for eligibility, seven were excluded based on a wrong study design (4), a wrong setting (3), a professional population (2), a paediatric population (1) or a wrong patient population (1), leaving 12 eligible for inclusion (Figure 1).

### 2.6 Quality appraisal

The JBI quality‐appraisal checklists for qualitative research, analytical cross‐sectional studies and quasi‐experimental studies (non‐randomized experimental studies) were used as well as the Mixed Methods Appraisal Tool, version 2018 (Hong et al., 2018), for one mixed‐methods study. Two researchers independently evaluated the quality of the included studies and any disagreements were resolved by discussion; if needed, a third researcher was consulted. No studies were excluded based on their quality‐appraisal scores.

### 2.7 Data extraction

Two researchers independently extracted data from the studies using a predefined Covidence form (Covidence, 2023). This template was systematically structured to capture key details, including the study's title, author(s), country of origin, aim, quality‐appraisal score, design, year of publication, data collection methods, data analysis methods, setting, population description, total number of participants, outcomes and main findings.

### 2.8 Synthesis

After the data were extracted from the identified studies' results sections, data‐based convergent synthesis was used to synthesize the data (Pluye & Hong, 2014). The variables from quantitative studies and the quantitative parts of the mixed‐methods studies were identified and qualitatively open‐coded (Hong et al., 2017). Thematic content analysis was used to integrate the findings from the original studies by breaking the information into codes and themes and grouping it accordingly (Vaismoradi et al., 2013).

## 3 RESULTS

### 3.1 Characteristics of the studies

The outline of the selected studies is presented separately (Table 1). Twelve included studies used four study designs: qualitative research (8), cross‐sectional studies (2), a non‐randomized experimental study (1) and mixed methods (1).

**TABLE 1:** Characteristics of the included studies.

Author(s), year, country, quality appraisal score	Aim of study	Study design	Data collection and analysis method	Setting and participants
Biasibetti et al., 2019, Brazil, QA 7/10	To analyse the perception of health professionals and companions/family about the development of communication for patient safety in paediatric hospitalizations	Qualitative research	Interviews, thematic content analysis	Paediatric clinical‐surgical units, health professionals, companions of hospitalized children (*n* = 138)
Bigani and Correia, 2018, USA, QA 7/10	To explore nurse, patient and family perceptions of change‐of‐shift bedside reports in the paediatric setting and describe specific safety concerns identified during change‐of‐shift handoff	Qualitative research	Interviews, thematic analysis	Paediatric units, nurses, patients and families (*n* = 40)
Buser et al., 2013, USA, QA 7/8	To determine whether parents of hospitalized children perceive that they have a role in preventing healthcare‐associated infections and whether they are willing to remind healthcare workers to perform hand hygiene, with and without an invitation	Cross‐sectional study	Structured interviews, descriptive and comparative analysis	Hospital setting, parents of hospitalized children (*n* = 115)
Costa et al., 2021, Brazil, QA 9/10	To describe the meaning attributed to the use of a game as a form of educational technology for the involvement of companions in paediatric patient safety	Qualitative research	Semi‐structured interviews, inductive content analysis	Hospital setting, companions of hospitalized children (*n* = 16)
Cox et al., 2017, USA, QA 8/8	To assess whether needing to watch over care predicts parent performance of recommended safety behaviours to reduce medication errors and healthcare‐associated infections	Cross‐sectional study	Survey, statistical analysis	Hospital setting, parents of hospitalized children (*n* = 170)
Franco et al., 2020, Brazil, QA 9/10	To determine the meaning attributed by family members to the health and safety of paediatric patients.	Qualitative research	Interviews, content analysis	Hospital setting, family members of hospitalized children (*n* = 18)
Hoffmann et al., 2019, Brazil, QA 9/10	To analyse patient safety incidents identified by caregivers of hospitalized children	Qualitative research	Interviews, thematic analysis	Hospital setting, caregivers of hospitalized children (*n* = 40)
Hoffmann et al., 2020, Brazil, QA 9/10	To determine the main safety incidents reported by relatives of patients hospitalized in paediatric units	Qualitative research	Interviews, thematic content analysis	Hospital setting, relatives of paediatric patients hospitalized in emergencies, infirmaries and intensive care centres (*n* = 91)
Khan et al., 2018, USA, QA 8/9	To determine whether medical errors, family experience and communication processes improved after implementation of an intervention to standardize the structure of healthcare provider–family communication on family‐centred rounds	Non‐randomized experimental study	Prospective, electronic patient medical database and adverse event data, statistical analysis	Paediatric inpatient units in seven North American hospitals, patients, parents or caregivers (*n* = 2148), nurses, medical students, residents
Lyndon et al., 2017, USA, QA 6/7	To describe parents' perspectives and likelihood of speaking up about safety concerns in the neonatal intensive care unit and identify barriers and facilitators to parents' speaking up	A mixed‐method study	Questionnaires, interviews, observations, qualitative and quantitative analyses	Neonatal intensive unit, parents of hospitalized newborns (*n* = 46 questionnaires and *n* = 14 interviews)
Ottosen et al., 2019, USA, QA 9/10	To determine how parents of neonates conceptualize patient safety in the neonatal intensive care unit	Qualitative research	Qualitative interviews, observations, thematic content analysis	Neonatal intensive care unit, parents of neonates in the NICU (*n* = 22)
Rodrigues et al., 2018, Brazil, QA 8/10	To analyse how parents identify patient safety in a neonatal unit	Qualitative research	Semi‐structured interviews, thematic content analysis	Neonatal intensive care unit, parents of children hospitalized in the neonatal unit (*n* = 23)

All 12 included studies were conducted between 2013 and 2023 in the United States (*n* = 6) or Brazil (*n* = 6). Eight studies were qualitative, two analytical cross‐sectional, one quasi‐experimental and one mixed‐method. In total, the studies included 3774 respondents, of whom 2789 were caregivers of hospitalized children, most of whom were their parents and over 18 years of age.

### 3.2 Quality appraisal

Table 1 presents the quality appraisal scores. One analytical cross‐sectional study and one mixed‐methods study (Cox et al., 2017; Lyndon et al., 2017) met all of the quality checklist requirements. The most common failure in the qualitative studies was a lack of a statement locating the researcher culturally or theoretically and the researcher's influence on the research was often not addressed. One quasi‐experimental study (Khan et al., 2018) lacked a control group, although it did provide a before‐and‐after comparison of the intervention. In one cross‐sectional study, the researchers had difficulties stating their strategy for addressing confounding factors (Buser et al., 2013). All included qualitative studies addressed data saturation in choosing their data samples. Despite not meeting all of the criteria, all studies received satisfactory grades enabling their inclusion in the study, as, in the researchers' view, they met crucial requirements and their value was not diminished. Table 2 summarizes the studies' findings.

**TABLE 2:** Findings of paediatric patient safety in hospital care from qualitative and quantitative studies.

Themes	Subthemes	Findings	References
Parental perceptions of inclusion in paediatric patient safety	Comfort in communication	Information provides security	Biasibetti et al. (2019)
			Bigani and Correia (2018)
			Ottosen et al. (2019)
		Expressing concerns increased after the intervention (18.2% [5.6%–45.3%] vs. 37.7% [17.6%–63.3%], *p* = 0.03)	Khan et al. (2018)
		Educational game as communication support	Costa et al. (2021)
		Willingness to participate in preventing healthcare‐associated infections (95% CI: 2.2–15.16; *p <* 0.0001)	Buser et al. (2013)
		Willingness to participate	Ottosen et al. (2019)
		Willingness to be informed	Hoffmann et al. (2019)
			Ottosen et al. (2019)
		Need for guidance: explicit invitation from healthcare workers is helpful (78%, *n =* 90)	Buser et al. (2013)
	Parental engagement	Watchful parents more likely to ask about a drug's name/dose (95% CI 1.3–7.4; *p <* 0.05)	Cox et al. (2017)
		Watchful parents more likely to verify drug/infusion accuracy (95% CI 2.1–9.9; *p <* 0.05)	Cox et al. (2017)
		Need for parent engagement	Franco et al. (2020)
			Ottosen et al. (2019)
		Parents' belief in their role to improve healthcare workers' hand hygiene (95% CI: 1.64–13.77; *p* = 0.004)	Buser et al. (2013)
		Parents' belief in the importance of their role	Ottosen et al. (2019)
			Rodrigues et al. (2018)
		Educational game supports parents' engagement	Costa et al. (2021)
		Family‐centred communication programme improves family engagement on rounds (55.6% [32.9%–76.2%] vs. 66.7% [43.0%–84.1%], *p* = 0.04)	Khan et al. (2018)

Abbreviations: CI, confidence interval; *p*, *p*‐value.

### 3.3 Paediatric patient safety in hospital care

**FIGURE 2:**
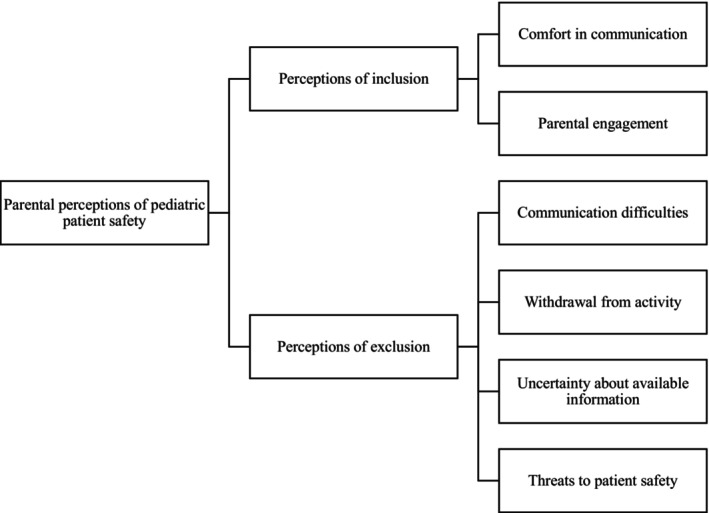
Thematic map of themes and subthemes.

The outcomes related to paediatric patient safety perceptions were grouped into two themes—parental perceptions of inclusion in paediatric patient safety and parental perceptions of exclusion from paediatric patient safety—and consisted of six subthemes (Figure 2): comfort in communication (Biasibetti et al., 2019; Bigani & Correia, 2018; Buser et al., 2013; Costa et al., 2021; Hoffmann et al., 2019; Khan et al., 2018; Ottosen et al., 2019), parental engagement (Buser et al., 2013; Costa et al., 2021; Cox et al., 2017; Franco et al., 2020; Khan et al., 2018; Ottosen et al., 2019; Rodrigues et al., 2018), communication difficulties (Franco et al., 2020; Hoffmann et al., 2019, 2020; Lyndon et al., 2017; Ottosen et al., 2019; Rodrigues et al., 2018), withdrawal from activity (Lyndon et al., 2017; Ottosen et al., 2019), uncertainty about available information (Hoffmann et al., 2019) and threats to patient safety (Franco et al., 2020; Hoffmann et al., 2019, 2020; Ottosen et al., 2019; Rodrigues et al., 2018) (Table 2).

#### 3.3.1 Comfort in communication

This subtheme highlighted the positive aspects of communication with healthcare workers and its influence on parents as well as troubles that parents faced when properly engaging in care during the hospitalization period.

Parents decidedly felt safest when they were included in the transmission of information between the healthcare professionals and given the opportunity, they were eager to be active participants. Being seen as valuable team members and playing a part in care were considered important (Biasibetti et al., 2019; Bigani & Correia, 2018; Khan et al., 2018; Ottosen et al., 2019), with examples of confusion coming from being given different medical opinions and having difficulty identifying which information should be followed (Hoffmann et al., 2020). Costa et al. (2021) demonstrated that communication shortcomings could be regulated by using new technology, such as informative games, to provide parents with the necessary knowledge that might be overlooked by medical staff (Costa et al., 2021). Parents show interest in participating in their hospitalized children's care when given instructions (Buser et al., 2013; Ottosen et al., 2019). However, the key to parental participation remains the possibility of receiving guidance from hospital staff, as they are wary of appearing rude. Moreover, being in the foreign setting of a hospital unit, parents recognize their need for information from the hospital staff to be able to participate in their children's care (Hoffmann et al., 2019; Ottosen et al., 2019). Accordingly, once instructions and a verbal invitation are given, parents are more willing to engage in safety‐oriented interventions (Buser et al., 2013).

#### 3.3.2 Parental engagement

The sub‐theme of parental engagement focused on the role parents play during hospitalization and their need to participate.

Parents perceive themselves as partners to hospital staff in pursuit of providing a safe environment for their children. They see their engagement as an obligation to ensure that no safety incidents occur (Franco et al., 2020), considering themselves vital and valuable participants in ensuring patient safety implementation during the hospital stay with their offspring (Buser et al., 2013;Ottosen et al., 2019; Rodrigues et al., 2018). The more protective the parents, the more likely they are to engage in ensuring that safety protocols are employed around their children. That includes asking and reminding healthcare professionals about hand hygiene and voicing their concerns around medication safety, correct doses and routes of admission (Cox et al., 2017).

Including parents in interventions may not change the overall experience, but it positively influences their comprehension of their surroundings and the information they receive, grants them a broader sense of being understood by healthcare professionals and provides a feeling of inclusion and freedom to voice potential concerns to staff (Khan et al., 2018). Providing parents with informative games helped them grasp the concept of patient safety, a position they might have in securing its implementation while encouraging them to be more engaged (Costa et al., 2021).

#### 3.3.3 Communication difficulties

The most common sub‐themes found throughout the included studies were difficulties regarding communication in the hospital setting and related obstacles. Of those, different findings emerged that focused on the need for inclusion and attention that parents of the hospitalized children voiced. The findings were: decision chaos, the need for information, the lack of relaying of information, anxiety about potential conflict and retaliation and the institutional approach.

Parents monitor any changes in their child's state, as well as the implementation of medical orders and their correctness, with numerous errors occurring because of the exclusion of parents from participation in the chain of decision‐making or not informing them in time about planned interventions (Franco et al., 2020; Hoffmann et al., 2019, 2020; Lyndon et al., 2017; Ottosen et al., 2019; Rodrigues et al., 2018). The insufficient communication and information transferal was perceived as an oversight, with a lack of space provided for an honest exchange of thoughts with medical personnel (Ottosen et al., 2019; Rodrigues et al., 2018). Parents pointed out their fearful feelings in relation to the process of obtaining information, being apprehensive about appearing as confrontational or undermining staff authority and uncertain of the possible consequences from the personnel. They were subjected to hostile attitudes and worried that their children might be exposed to the consequences of their “speaking up” and might suffer worse medical care (Hoffmann et al., 2019; Lyndon et al., 2017).

Parents observed attention paid by the hospital staff to the children and how the bureaucratic assignments sometimes took precedence over watching their patients, the interactions of hospital staff with paediatric patients, their attentiveness towards the children and their needs and healthcare professionals' responsiveness to their surroundings (Ottosen et al., 2019). Companions noted that they were not always allowed to accompany their children into the rooms where the procedures, such as drawing blood, were performed (Hoffmann et al., 2019).

#### 3.3.4 Withdrawal from activity

This segment highlighted the specific situation in which parents found themselves when accompanying their children in the hospital environment, as it involved changes to the daily routine of both children and their parents and often required the relinquishment of some of all parental tasks regarding the ill child. The environment in which they found themselves was usually unfamiliar, crowded and loud, forcing them to relinquish control over their authority and surroundings (Lyndon et al., 2017). Once the situation was established and they could establish a partnership with clinicians and staff, they wanted to engage in the provision of care and reclaim as much responsibility as possible in the given circumstances (Ottosen et al., 2019).

#### 3.3.5 Uncertainty about available information

This sub‐theme addressed concerns about the faulty guidelines provided by the institutions. Parents understood the need to abide by the rules of the hospital, although they noted that following safety measurements might prove troublesome and that the instructions given by the hospital were sometimes deficient or incomplete. Parents voiced their concerns that the instructions provided by the hospital about the use of hospital infrastructure, maintaining hand hygiene, isolation of infectious patients and the use of protective equipment were either imprecise or nonexistent (Hoffmann et al., 2019).

#### 3.3.6 Threats to patient safety

This sub‐theme comprises the points made by parents about the situations they observed during the hospitalization of their children that posed a risk to their health and wellbeing. It was split into inadequate adaptation to children, epidemiological risk, hygiene risk, concern about medication, infrequent patient identification and security control.

Patient safety was perceived as an institutional and professional obligation, with parents identifying several shortcomings in securing its implementation (Franco et al., 2020; Hoffmann et al., 2019, 2020; Ottosen et al., 2019; Rodrigues et al., 2018). The most mentioned were deficiencies in adherence to drug safety, with cases of missed doses of medication, incorrect doses or mistaking patients being reported. A lack of compliance with hygiene was expressed, including the selective use of disinfectants, gloves and other protective equipment (Hoffmann et al., 2020; Rodrigues et al., 2018). Parents mentioned confusion around the unit organization as well as an unsafe—in parents' view—approach to rooming children with contagious diseases with those suffering from other ailments (Hoffmann et al., 2019; Ottosen et al., 2019; Rodrigues et al., 2018). A lack of proper equipment (e.g., beds and barriers) resulted in the perception of reduced safety, with notes about inadequate adaptation of hospitals for impaired children (Franco et al., 2020; Hoffmann et al., 2019). Units were described as insecure due to a lack of identification verification at the entrances, resulting in parental anxiety about who might enter the premises and be in contact with their offspring (Hoffmann et al., 2019; Ottosen et al., 2019; Rodrigues et al., 2018), as well as insufficient patient verification during the shift (Hoffmann et al., 2019).

## 4 DISCUSSION

Research about perspectives on paediatric patient safety was dominated by professional views, with limited sources considering the parental point of view. Such a knowledge gap is concerning in itself, but it is all the more troubling, as it creates an impression that staff still do not consider parents as valuable partners during medical treatment. Examples of both inclusion and exclusion practices in paediatric patient safety were found, however, showcasing that there are available solutions to enhance the experience of paediatric hospitalization.

The study's principal discovery is that parents are not only willing but also prepared to actively engage in the safety protocols concerning their children's hospital care. This aligns with the findings of Lyndon et al. (2014), which corroborate parents' readiness to participate in their child's hospital care. It is the healthcare facilities' responsibility to enable them to engage as equal partners in ensuring the implementation of safety solutions (Lyndon et al., 2014). This coincides with previous research indicating that providing parents with the means to actively participate not only allows them to get used to the situation but also allows for the observation of family dynamics and responses to possible gaps in knowledge or behaviour. Using games as educational tools could be considered as one way to support parents during their hospital stay, as such games could provide them with answers to some concerns and questions about hospitalization. Providing games as an educational tool gives the opportunity to hold conversations about challenging issues while playing, minimizing stressors directed towards parents while providing access to information regarding the hospital stay (de Gonçalves et al., 2020; Fernandes et al., 2018).

Parents have emphasized their willingness to participate in decision‐making and aid the healthcare professionals. Such sentiments coincide with the established evidence that mobilizing parental engagement and increasing parental participation is beneficial for both the adults and children and may help prevent potential adverse events. It is advisable to enable them to increase their participation in care as well as to ensure that they realize their rights and encourage their involvement (de Melo et al., 2014; Jones et al., 2021; Mueller et al., 2019; Weingart et al., 2013). Parental participation in care has been proven beneficial in recognizing and documenting preventable adverse events that are often missed by medical staff. Previous research indicates that it is worth considering the greater participation of families in the hospital patient safety monitoring system. Emphasis should be placed on thoroughly informing families about treatment plans and encouraging them to report any errors they notice, as there is a discernible link between inadequate communication and the occurrence of mistakes. When creating interventions involving families, ways to reduce the distance between healthcare professionals and parents and considering using the hospitals' existing safety monitoring systems should also be considered (Khan et al., 2016, 2017). Because the paediatric setting is more susceptible to errors, especially drug‐supply errors, healthcare organizations should consider implementing proactive risk‐management methods that would not only improve patient safety but also provide preventive measures against said errors (D'Errico et al., 2022).

Concerns and comments related to communication were a primary theme throughout the included papers. Parents indicated their feelings of being lost and ignored during hospitalization. The literature acknowledges that they are often exposed to sadness, stress and a sense of abandonment. Leaving their everyday, familiar lives can undoubtedly destabilize their authority (Gurtovenko et al., 2021; Toledano‐Toledano & Luna, 2020).

Despite frequent exclusion from the information cycle, parents monitored the interventions to which their children were subjected. Their apprehension about confronting their concerns and remarks regarding care with the staff for fear of their retribution was significant; however, previous studies show that insufficient communication may be a result of increased levels of stress among both parents and medical staff, as well as anxiety among the healthcare professionals themselves about pointing out each other's mistakes within the same profession or between them (e.g., between doctors and nurses). It often results from previous unpleasant experiences in this regard and fears of being perceived as uneducated or unprofessional, even though it might affect the quality of care and patient safety. Families often share similar feelings, wanting to avoid being labelled as troublemakers (Bell et al., 2018; Gluyas, 2015; Okuyama et al., 2014).

Parents acknowledge the feeling of giving up control in the hospital setting. As previously reported, this is because hospital wards operate on completely different principles than regular life and their surroundings vastly contrast with typical everyday settings, with sounds and people with whom one does not usually come into contact (Bevan et al., 2019; Stickland et al., 2016). Support from the healthcare professionals helps parents regain their authority, but they should be provided with the opportunity to make decisions and engage within their capabilities and willingness (Buetow et al., 2013; Çamur & Sarıkaya Karabudak, 2021).

Despite parents' reported inhibitions about “disturbing” staff and anxiety about foreign environment and the often unknown or unsteady health situation of their child, parents are ready to regain, at least partially, their agency. Previous research indicates that staff involvement is crucial both in providing explanations of procedures and by monitoring parents' progress as they begin to participate in their child's care again. Accommodating the parental need to prepare and evaluate their abilities increases their self‐confidence and benefits the overall assessment of their child's hospitalization period (Çamur & Sarıkaya Karabudak, 2021; Dadlez et al., 2018; Lam et al., 2014).

### 4.1 Strengths and limitations

This systematic review was based on a carefully followed protocol. At least two researchers were engaged in every phase of the review process, with a search strategy designed with guidance provided by an information specialist. To ensure the relevance of the included data, only studies from the past 10 years were included.

Studies needed to be available in English, so there is a risk that other otherwise‐admissible studies were omitted. The eligible studies were based only in the United States and Brazil and potential cultural differences and country‐specific procedures and regulations should be considered when reviewing the obtained results. There were no major differences among the eligible studies and their conclusions; however, to get reliable results concerning parental perceptions of paediatric patient safety in a hospital setting, it will be necessary to conduct larger studies with larger samples and a wider geographical reach, with intervention studies providing information on factors affecting those perceptions.

## 5 CONCLUSIONS

In paediatric nursing, the development and execution of strategies that actively involve parents in the care of their hospitalized children should be prioritized. This collaborative approach is crucial for mitigating risks and preventing errors. By amplifying parental participation in both the caregiving and decision‐making processes, we can ensure more comprehensive and safer care for children. Parents are willing to engage but require help from staff to ensure the safety of paediatric patients together. Hospitals and healthcare organizations need to consider implementing solutions and interventions that include the parents of hospitalized children in providing care and preventing adverse events and errors, such as increasing their involvement in care and allowing them to participate in the decision‐making process concerning their child.

## AUTHOR CONTRIBUTIONS

All authors made substantial contributions to conception and design or acquisition of data or analysis and interpretation of data, involved in drafting the manuscript or revising it critically for important intellectual content. All authors given final approval of the version to be published. Each author participated sufficiently in the work to take public responsibility for appropriate portions of the content, agreed to be accountable for all aspects of the work in ensuring that questions related to the accuracy or integrity of any part of the work are appropriately investigated and resolved.

## FUNDING INFORMATION

This research received no specific grant from any funding agency in the public, commercial or not‐for‐profit sectors.

## CONFLICT OF INTEREST STATEMENT

No conflict of interest has been declared by the authors.

## PEER REVIEW

The peer review history for this article is available at https://www.webofscience.com/api/gateway/wos/peer‐review/10.1111/jan.16361.

## Supporting information


Appendix S1.

Data S1.

## Data Availability

The data supporting the findings of this study are available in the supplementary material of this article. All data utilized in the submitted manuscript have been lawfully acquired in accordance with The Nagoya Protocol on Access to Genetic Resources and the Fair and Equitable Sharing of Benefits Arising from Their Utilization to the Convention on Biological Diversity.
