# Supplementary material for: Parents' perceptions of patient safety in paediatric hospital care—A mixed‐methods systematic review
Source: J Adv Nurs. 2024 Aug 9;81(9):5291–303. doi: 10.1111/jan.16361 (PMC12371830; doi:10.1111/jan.16361)
Supplement: Supplementary file 1 — Appendix S1. [file JAN-81-5291-s001.docx]

**Appendix 1**

**PubMed 15 results accessed on 22 July 2023**

**Search: (("patient safety"[Title]) AND ((mother*[Title/Abstract] OR father*[Title/Abstract] OR caregiver*[Title/Abstract] OR care-giver*[Title/Abstract] OR famil*[Title/Abstract] OR guardian*[Title/Abstract]))) AND ((child*[Title/Abstract] OR daughter[Title/Abstract] OR son[Title/Abstract] OR p*ediatric[Title/Abstract] OR offspring[Title/Abstract] OR minor*[Title/Abstract] OR infant*[Title/Abstract] OR neonate*[Title/Abstract] OR teen*[Title/Abstract] OR adolescent*[Title/Abstract] OR toddler*[Title/Abstract])) AND ((experience*[Title/Abstract] OR perception*[Title/Abstract] OR perspective*[Title/Abstract] OR view*[Title/Abstract] OR insight*[Title/Abstract] OR impression*[Title/Abstract] OR image*[Title/Abstract] OR observation[Title/Abstract])) AND (experience*[Title/Abstract] OR perception*[Title/Abstract] OR perspective*[Title/Abstract] OR view*[Title/Abstract] OR insight*[Title/Abstract] OR impression*[Title/Abstract] OR image*[Title/Abstract] OR observation*[Title/Abstract] OR opinion*[Title/Abstract] OR attitude*[Title/Abstract]) Filters: in the last 10 years**

**PubMed 29 results accessed on 29 July 2023**
**Search: (("patient safety"[Title]) AND ((pediatric[Title] OR paediatric[Title] OR child*[Title]))) AND ((family OR parent* OR "care-giver*" OR caregiver*)) Filters: in the last 10 years**

**Scopus 41 results accessed on 22 July 2023**

(TITLE("patient safety") AND TITLE-ABS-KEY(mother OR father OR caregiver* OR "care-giver*" OR famil* OR guardian*) AND TITLE-ABS-KEY(child* OR daughter OR son OR p*ediatric OR offspring OR minor* OR infant* OR neonate* OR teen* OR adolescent* OR toddler*) AND TITLE-ABS-KEY(experience* OR perception* OR perspective* OR view* OR insight* OR impression* OR image* OR observation* OR opinion* OR attitude*)) AND PUBYEAR > 2012 AND PUBYEAR < 2024

**Scopus 62 results accessed on 29 July 2023**

( TITLE ( "patient safety" AND pediatric OR paediatric OR child* ) AND ALL ( family OR parent* OR "care-giver*" OR caregiver* ) ) AND PUBYEAR > 2012 AND PUBYEAR < 2024

**ScienceDirect 367 results accessed on 22 July 2023**

[Find articles with these terms]

(mothers OR fathers OR caregivers OR family OR families OR familial OR guardians)

[Years]

2013-2023

[Title, abstract or author-specified keywords]

"patient safety" AND (experiences OR perceptions OR perspectives OR views OR impressions OR observations OR opinions OR attitudes)

[References ]

(children OR daughter OR son OR pediatric OR offspring OR infants OR neonates OR adolescents

**ScienceDirect 323 results accessed on 29 July 2023**

[Find articles with these terms]

(family OR parent OR parents OR parental OR "care-giver" OR "care-givers" OR caregiver OR caregivers)

[Years]

2013-2023

[Title, abstract or author-specified keywords]

"patient safety" AND (pediatric OR paediatric OR child OR children)

**The Cochrane Library 4 reviews accessed on 22 July 2023**

## (“patient safety”) in Title Abstract Keyword AND (experience* OR perception* OR perspective* OR view* OR insight* OR impression* OR image* OR observation* OR opinion* OR attitude*) in Title Abstract Keyword AND (mother OR father OR caregiver* OR (care NEXT giver*) OR famil* OR guardian*) in Title Abstract Keyword AND (child* OR daughter OR son OR p*ediatric OR offspring OR minor* OR infant* OR neonate* OR teen* OR adolescent* OR toddler*) in Title Abstract Keyword - with Cochrane Library publication date Between Jan 2013 and Dec 2023 (Word variations have been searched)

**The Cochrane Library 5 reviews accessed on 29 July 2023**

## (“patient safety”) AND (pediatric OR paediatric OR child*) in Title Abstract Keyword AND (family OR parent* OR (care NEXT giver*) OR caregiver*) in All Text - with Cochrane Library publication date Between Jan 2013 and Dec 2023 (Word variations have been searched)

**Wiley 27 results accessed on 22 July 2023**

[Title] (“patient safety”) AND experience* OR perception* OR perspective* OR view* OR insight* OR impression* OR image* OR observation* OR opinion* OR attitude*

[Abstract] mother OR father OR caregiver* OR "care-giver*" OR famil* OR guardian*

AND (child* OR daughter OR son OR p*ediatric OR offspring OR minor* OR infant* OR neonate* OR teen* OR adolescent* OR toddler*)

Publication date: Custom range 2013-2023

**Wiley 10 results accessed on 22 July 2023**

"("patient safety") AND (pediatric OR paediatric OR child*)" in Title and "(family OR parent* OR "care-giver*" OR caregiver*)"

Publication date: Custom range 2013-2023
